# Field Observations and Genetic Characterization of Sheep-Associated Malignant Catarrhal Fever in Egypt, 2018

**DOI:** 10.3390/vetsci7040201

**Published:** 2020-12-11

**Authors:** Sahar Abd El Rahman, Ahmed Ateya, Mohamed El-Beskawy, Kerstin Wernike, Bernd Hoffmann, Michael Eschbaumer

**Affiliations:** 1 Department of Virology, Faculty of Veterinary Medicine, Mansoura University, Mansoura 35516, Egypt; sahar_virol@mans.edu.eg; 2 Department of Animal Husbandry and Development of Animal Wealth, Faculty of Veterinary Medicine, Mansoura University, Mansoura 35516, Egypt; ahmed_ismail888@yahoo.com; 3 Department of Animal Medicine, Faculty of Veterinary Medicine, Matrouh University, Matrouh 51744, Egypt; melbeskawy@gmail.com; 4 Institute of Diagnostic Virology, Friedrich-Loeffler-Institut, 17493 Greifswald-Insel Riems, Germany; kerstin.wernike@fli.de (K.W.); bernd.hoffmann@fli.de (B.H.)

**Keywords:** sheep-associated malignant catarrhal fever, ovine gammaherpesvirus 2, ORF27, gp48, Egypt

## Abstract

Ovine gammaherpesvirus-2 (OvHV-2) causes a lethal disease in cattle and some wild ruminants called malignant catarrhal fever (MCF), which affects the epithelial and lymphoid tissues of the respiratory and digestive tracts and has an important impact on the livestock industry. In this study, MCF was diagnosed in 18 of 427 cattle from different sites in Egypt by its typical clinical signs, found in all 18 animals: corneal opacity, fever, erosions in the buccal cavity, lymphadenitis, and purulent nasal discharge. All affected cattle had been reared in contact with clinically inconspicuous sheep. Of the 18 clinically ill cattle, 13 succumbed to the disease, resulting in estimated morbidity and case fatality rates of 4.2% and 72.2%, respectively. Five samples collected from the affected cattle were positive for OvHV-2 by real-time PCR and were used for sequencing of an 832-bp fragment of the ORF27/gp48 gene. The ORF27 nucleotide sequence of all Egyptian samples was identical, but distinct from viruses found in other parts of Africa and the Mediterranean.

## 1. Introduction

Malignant catarrhal fever (MCF) is a disease caused by a group of herpesviruses of the genus *Macavirus* in the family *Alloherpesviridae*, subfamily *Gammaherpesvirinae*. Its causative viruses exist in nature as lifelong and usually subclinical infections in reservoir hosts. The viruses have coevolved with their reservoirs over long periods of time and do no longer cause disease in these hosts, but they can be lethal when they are transmitted to animals of other species to which they are poorly adapted [1].

Several epidemiologic forms are distinguished based on the reservoir species, most notably sheep-associated MCF caused by ovine gammaherpesvirus 2 (OvHV-2) and wildebeest-associated MCF caused by alcelaphine gammaherpesvirus 1 (AlHV-1) [1]. Wildebeest-associated MCF in African cattle is a seasonal disease and occurs most frequently during wildebeest calving season, driven by virus transmission from young wildebeest calves [2]. By contrast, sheep-associated MCF occurs all year. Its incidence is moderately increased in the spring, coinciding with the lambing season in temperate climates, but this increase is not caused by the lambing itself. Newborn lambs are not infected and do not shed the virus [3], nor is there an increase in virus shedding from ewes during lambing [4]. Like other herpesviruses, the viruses causing MCF are not very stable in the environment [5]. They are shed by their reservoir hosts in nasal secretions [4,6], and clinically susceptible species acquire the virus through inhalation, ingestion of virus-laden secretions, or possibly through contaminated feed and water [1]. Transmission of OvHV-2 from sheep to bison over significant distances has been documented [7], but it is most efficient when reservoir hosts and animals of clinically susceptible species are in close contact. AlHV-1 and OvHV-2 are not transmitted by natural means from one clinically susceptible host to another and MCF-affected animals do not pose a risk for their mates [8,9]. Therefore, disease caused by AlHV-1 is restricted to those parts of Africa and zoological collections in the rest of the world where wildebeest (*Connochaetes* spp.) are present. Sheep-associated MCF, however, occurs worldwide wherever sheep are being reared.

In cattle, MCF is a significant and often lethal disease characterized by lymphoproliferation and inflammation, predominantly in mucosal surfaces and blood vessels (arteritis) [10]. After an incubation period of 2–10 weeks or longer, clinical signs usually begin with depression and high fever, followed by oculonasal discharge due to keratoconjunctivitis and rhinitis. Necrosis and ulceration of mucous membranes, including the oral mucosa, intestinal mucosa, and urinary tract, are often observed [10]. Taken together, the clinical picture allows a presumptive diagnosis of MCF, but other conditions that cause oral lesions, such as mucosal disease (a sequela of persistent infection with bovine viral diarrhea virus; BVDV), infectious bovine rhinotracheitis (caused by bovine alphaherpesvirus 1; BoHV-1), bluetongue, foot-and-mouth disease (FMD), and vesicular stomatitis (in the Americas), must also be considered and ruled out by laboratory testing if possible [11]. Supporting evidence for MCF can be collected by histopathological examination, but the method of choice to confirm the diagnosis is the detection of viral DNA in blood (buffy coat) or tissue from lymph nodes, lungs, or spleen [5]. It is important to note that the mucosal lesions of MCF-affected animals are caused by immunopathological processes and not by virus replication [12]; therefore, the viral load in lesion material and secretions is generally very low [13]. The amplification of viral DNA from clinical samples by PCR is also necessary for the genetic characterization of circulating strains for epidemiological studies [14]. Despite reports to the contrary [15], there currently is no productive cell culture system for OvHV-2 [13].

In Europe and North America, MCF is considered to be of minor importance to the cattle industry, and it is a concern mostly in bison, farmed deer, and zoological collections [10]. Its impact in developing countries, on the other hand, is not well studied and may be considerable depending on herd structure and husbandry practices [14]. OvHV-2 was previously reported to cause sheep-associated MCF outbreaks in Egyptian cattle and buffalo in 2010 [16] and 2012/2013 [17]. In this study, we have investigated a more recent occurrence of the disease in the Delta region of Egypt in 2018.

## 2. Materials and Methods

### 2.1. Field Study

Between September and December of 2018, five herds of cattle in the Delta region of Egypt (see Table 1) were examined clinically according to Jackson and Cockcroft [18]. In all herds, cattle were reared together with sheep and goats and shared the same grazing. The locations of the herds were between 25 and 40 km apart.

Overall, 27 samples (18 buffy coat, 1 whole blood, 3 plasma, 3 serum, and 2 saliva samples) were taken from cattle with clinical signs and submitted for laboratory confirmation of OvHV-2 infection and exclusion of other relevant infectious diseases. No ethical approval was required for the study because this is a field survey based on diagnosis of natural infection with catarrhal fever and the samplings have been performed by veterinarians.

### 2.2. Nucleic Acid Extraction and Real-Time PCR

Nucleic acids were extracted from 100 µL of each sample with the NucleoMag VET kit (Macherey-Nagel, Düren, Germany) on a KingFisher Flex Magnetic Particle Processor (Thermo Fisher Scientific, Waltham, MA, USA) following the manufacturers’ instructions. The extracted nucleic acids were tested for AlHV-1 [19] and OvHV-2 [20] DNA by real-time PCR using the PerfeCTa qPCR ToughMix (Quantabio, Beverly, MA, USA) in a CFX96 thermocycler (Bio-Rad, Hercules, CA, USA) as well as for FMD virus [21], BVDV [22], and BoHV-1 [23] as previously described. A lymphoid tissue preparation from an Italian case of sheep-associated bovine MCF from 2007 (record no. D35/07; kindly provided by Dr. Horst Schirrmeier) was used as a positive control for the OvHV-2 PCR. Appropriate controls for nucleic acid extraction and PCR were used throughout.

### 2.3. Sequencing, Nucleotide Sequence Alignments, and Phylogenetic Analysis

Nucleic acid preparations that were positive in the OvHV-2 real-time PCR were used for sequencing using the primers described by Doboro et al. [14]. A 999-bp fragment of the OvHV-2 genome partially overlapping the ORF27 and ORF29 genes [24] was amplified using the QuantiTect Multiplex PCR NoROX Kit (Qiagen, Hilden, Germany). PCR products were screened by agarose gel electrophoresis, bands of the expected size were excised, the DNA was purified and submitted to Eurofins, Constance, Germany, for Sanger sequencing.

All sequence alignments in this study were performed with MUSCLE [25] 3.8.425 in Geneious Prime 2019.2.3 (https://www.geneious.com/home/). The evolutionary history was inferred using the Maximum Likelihood method and Kimura 2-parameter model [26] in MEGA X ([27]; https://www.megasoftware.net/). Statistical support for nodes in the dendrogram was obtained by bootstrapping [28] (1000 replicates); only values ≥50% are shown.

## 3. Results and Discussion

### 3.1. Clinical Disease

Among the 5 herds, 18 cattle were found with conspicuous clinical signs. Depression and anorexia, fever between 40 and 42 °C, enlargement of peripheral lymph nodes, corneal opacity, congestion of capillaries of the eyes, lacrimation and photophobia, erosive stomatitis, and nasal and ocular discharges, which progressed from serous to purulent exudates, were found in all 18 animals.

**Figure 1:**
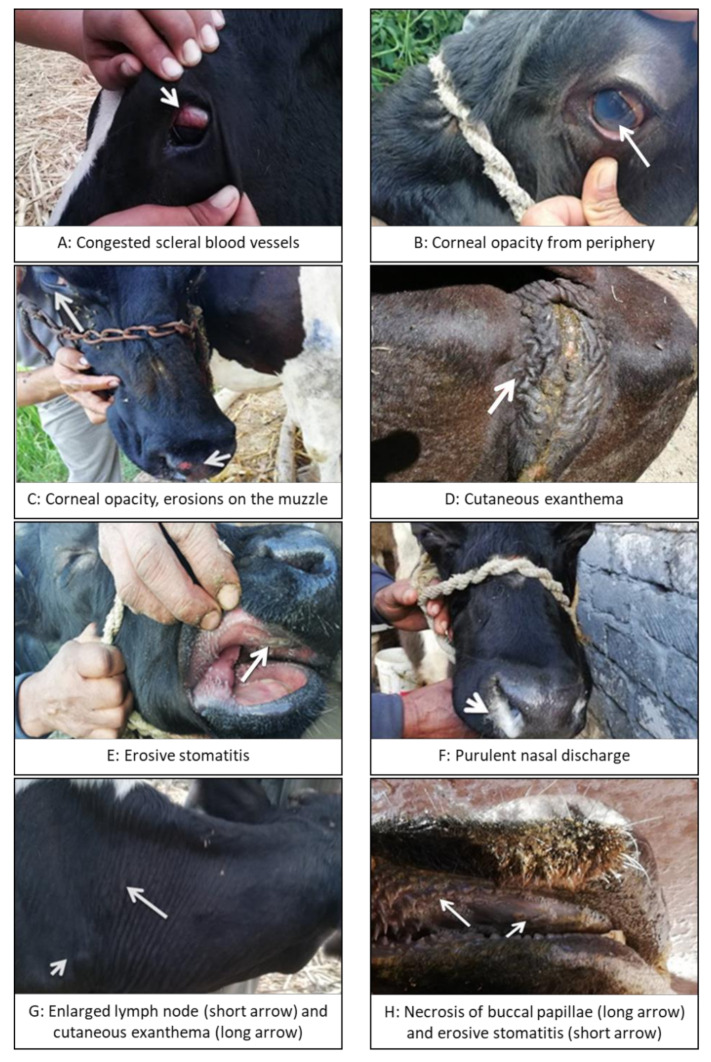
Clinical signs in malignant catarrhal fever (MCF)-affected cattle. Gloves should be worn at all times when attending to diseased animals.

Further signs seen in some of the animals included inflammation of buccal papillae, intense hyperemia, and multifocal or diffuse necrosis of the oral mucosa (usually on the lips, gums, and hard and soft palate), peeling of the muzzle in addition to cutaneous exanthema and dermatitis, red-tinged urine, and hemorrhagic diarrhea (see Table 2 and Figure 1).

All diseased cattle were females of 4–5 years, which also constituted the majority of animals in the examined herds. Over the course of the disease, 13 of the animals became recumbent and ultimately expired between 1 week and 10 days after the first appearance of clinical signs. The overall morbidity and case fatality rates were 4.2% (18 of 427 animals) and 72.2% (13 of 18 animals), respectively, with little variation between herds (see Table 3).

### 3.2. Virus Detection, Nucleotide Sequence Determination, and Phylogenetic Analysis

Five of the 27 samples collected from the affected cattle were positive for OvHV-2 by real-time PCR with quantification cycle (C_q_) values between 31 and 36 (see Table 4), but none were positive for AlHV-1, FMDV, BVDV, or BoHV-1. The presumptive clinical diagnosis of MCF was confirmed in only three of 18 animals by detection of OvHV-2 DNA in blood. It is important to note, however, that the high C_q_ values in these samples and the lack of OvHV-2 detection in the other animals could be due to sample deterioration in transit from the collection sites in Egypt to the laboratory in Germany. The tested samples also included saliva, which in MCF-affected animals is unlikely to contain detectable OvHV-2 DNA, but is an important sample to rule out FMD or mucosal disease.

Four partial ORF27 coding sequences of 832 bp (missing 50 nucleotides at the 5′ end) were obtained from the PCR-positive samples. No sequence data were obtained with any of the other primer pairs described by Doboro et al. [14]. All 4 ORF27 sequences were identical and distinct from the sequence of the OvHV-2 sample D35/07 used as a positive control for the PCR. The nucleotide sequence of the Egyptian samples has been uploaded to the NCBI GenBank (accession no. MT821904).

**Figure 2:**
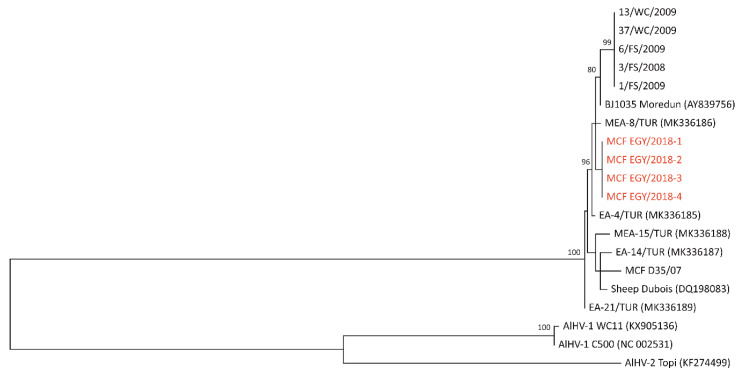
Basic ORF27 phylogeny. Egyptian sequences from this study are highlighted in red. The South African FS (Free State) and WC (Western Cape) sequences are from Doboro et al. [14], Turkish MEA and EA sequences are from Turan et al. [29]. Both datasets are based on laboratory-confirmed cases of MCF. BJ1035 is the first complete ovine gammaherpesvirus 2 (OvHV-2) genome published by Hart et al. [24], which was ultimately derived from a Scottish cow with sheep-associated MCF [12], and DQ198083 is a composite sequence of viral DNA enriched from nasal secretions of 13 sheep at the U.S. Sheep Experiment Station in Dubois, ID, USA, previously published by Taus et al. [30]. The D35/07 sequence is from a case of bovine sheep-associated MCF in Italy in 2007.

No ORF27 sequences from earlier studies in Egypt were available. A BLASTn search of the GenBank database (https://blast.ncbi.nlm.nih.gov/Blast.cgi) returned 7 OvHV-2 nucleotide sequences with significant similarity to the Egyptian sequences from this study (percent identity from 99.40% to 98.32%), as well as 2 unique AlHV-1 sequences (73.60% and 73.29%) and one AlHV-2 sequence (65.75%). The OvHV-2 sequences were downloaded, aligned, and trimmed to match the Egyptian sequences. The ORF27 sequence data published by Doboro et al. [14] (5 identical sequences) were extracted from the article and added manually. A maximum-likelihood phylogenetic tree of the 20 ORF27 nucleotide sequences included in this study is shown in Figure 2.

The ORF27 gene of OvHV-2 encodes a putative 35 kDa glycoprotein that is present in all gammaherpesviruses [31] and is referred to as gp48 based on earlier work with murine gammaherpesvirus 68 [32]. Alignments of the 10 unique ORF27/gp48 nucleotide and protein sequences (Figure S1) and pairwise distance matrices (Tables S1 and S2) are included in the Supplementary Material for this article. Based on the ORF27 nucleotide sequence, the Egyptian OvHV-2 samples were most similar to the BJ1035 reference strain (99.4% nucleotide identity) and three of the Turkish samples MEA-8, EA-4, and EA-21 (99.4%, 99.3%, and 99.0%, respectively; see Table S1). Among the ORF27 nucleotide sequences available for this study, the 5 identical South African sequences published by Doboro et al. [14] were most divergent (95.6–97.5% nucleotide identity), with several out-of-frame deletions creating considerable amino acid differences in some regions of the protein (Figure S1). The South African viruses only had 86.6% (with MCF D35/07) to 89.1% (with EA-4/TUR) amino acid identity with the other viruses in the study, considerably lower than the other viruses among themselves (96.4–99.6% identical amino acids) (Table S2). The gp48 protein sequence of the Egyptian strains only differed by 1 amino acid from the reference strain BJ1035, but by 31 from the South African viruses. This supports the earlier claim that the South African viruses constitute a separate genotype [14]. They are, however, clearly ovine gammaherpesviruses. The closest AlHV-1 sequence (reference strain C500, accession no. NC_002531) only had a nucleotide identity of 58.4–59.3%, and an amino acid identity of 45.0–49.3% (see Tables S1 and S2) with the OvHV-2 sequences in this study, including the South African sequences.

Based on the limited ORF27 sequence data that were available for this study, it is not possible to conclusively define further genotypes. It is evident, however, that the OvHV-2 strain identified in Egypt in 2018 is less closely related to South African strains than to viruses identified in other regions, including Eastern Turkey. Further research is required to provide a clearer picture of the ORF27 phylogeny of OvHV-2 in Africa and around the world [14,29].

## 4. Conclusions

The outbreaks of MCF in the Delta region of Egypt in 2018 were likely all caused by the same strain of OvHV-2. This suggests that the disease originated in the sheep that were reared together with the affected cattle, which is still common practice in Egypt. To reduce the incidence of MCF in Egyptian cattle, it is recommended to keep them separate from sheep at all times. All clinical suspicions of MCF must be supported by laboratory testing to rule out clinically similar conditions such as mucosal disease, infectious bovine rhinotracheitis, and FMD.

## Figures and Tables

**Table 1:** Sex and age distribution in cattle herds and numbers of coreared small ruminants.

	Cattle					Small Ruminants ^1^	
	Sex		Age				
Herd	Female	Male	<1 Year	1–3 Years	3–5 Years	Sheep	Goats
A	33	2	10	6	19	6	4
B	64	3	15	18	34	25	13
C	137	4	20	32	89	21	8
D	132	3	38	30	67	14	9
E	47	2	12	10	27	7	4
Total	413	14	95	96	236	73	38

^1^ All sheep were Ossemi breed, and all goats were Baladi breed.

**Table 2:** Clinical signs seen in cattle in this study.

Animal	Herd	Fever	Corneal Opacity	Erosions in Oral Cavity	Enlarged Lymph Nodes	Purulent Nasal Discharge	Hemorrhagic Diarrhea	Cutaneous Exanthema	Red-Tinged Urine	Peeling Skin on Muzzle
1	A	Yes	Yes	Yes	Yes	Yes	No	Yes	Yes	Yes
2	B	Yes	Yes	Yes	Yes	Yes	Yes	Yes	No	No
3	B	Yes	Yes	Yes	Yes	Yes	No	Yes	No	Yes
4	C	Yes	Yes	Yes	Yes	Yes	Yes	No	No	No
5	C	Yes	Yes	Yes	Yes	Yes	Yes	No	No	No
6	C	Yes	Yes	Yes	Yes	Yes	No	No	Yes	No
7	C	Yes	Yes	Yes	Yes	Yes	No	No	No	No
8	C	Yes	Yes	Yes	Yes	Yes	No	No	No	No
9	C	Yes	Yes	Yes	Yes	Yes	Yes	No	No	No
10	C	Yes	Yes	Yes	Yes	Yes	Yes	No	Yes	Yes
11	D	Yes	Yes	Yes	Yes	Yes	Yes	No	No	No
12	D	Yes	Yes	Yes	Yes	Yes	No	No	No	No
13	D	Yes	Yes	Yes	Yes	Yes	Yes	No	No	No
14	D	Yes	Yes	Yes	Yes	Yes	No	No	No	No
15	D	Yes	Yes	Yes	Yes	Yes	No	No	Yes	Yes
16	D	Yes	Yes	Yes	Yes	Yes	Yes	No	Yes	Yes
17	E	Yes	Yes	Yes	Yes	Yes	Yes	Yes	Yes	Yes
18	E	Yes	Yes	Yes	Yes	Yes	Yes	No	No	Yes
Total (n)		18	18	18	18	18	10	4	6	7
% of animals		100	100	100	100	100	56	22	33	39

**Table 3:** Morbidity and case fatality rates by herd.

Herd	Animals Examined	Diseased Animals	Morbidity Rate	Deaths	Case Fatality Rate
A	35	1	2.9%	1	100.0%
B	67	2	3.0%	2	100.0%
C	141	7	3.5%	5	71.4%
D	135	6	3.0%	4	66.7%
E	49	2	4.1%	1	50.0%
Total	427	18	4.2%	13	72.2%

**Table 4:** C_q_ values of the ovine gammaherpesvirus-2 (OvHV-2) PCR for samples from clinically affected cattle.

Animal	Herd	Buffy Coat	Whole Blood	Plasma	Serum	Saliva
1	A	No C_q_	No C_q_	n.s.	No C_q_	n.s.
2	B	31.03	n.s.	No C_q_	n.s.	n.s.
3	B	No C_q_	n.s.	n.s.	n.s.	n.s.
4	C	No C_q_	n.s.	No C_q_	n.s.	n.s.
5	C	No C_q_	n.s.	n.s.	n.s.	n.s.
6	C	No C_q_	n.s.	n.s.	n.s.	No C_q_
7	C	No C_q_	n.s.	n.s.	n.s.	n.s.
8	C	No C_q_	n.s.	n.s.	n.s.	n.s.
9	C	No C_q_	n.s.	n.s.	n.s.	n.s.
10	C	33.17	n.s.	n.s.	35.99	n.s.
11	D	No C_q_	n.s.	n.s.	n.s.	No C_q_
12	D	No C_q_	n.s.	n.s.	n.s.	n.s.
13	D	No C_q_	n.s.	n.s.	n.s.	n.s.
14	D	No C_q_	n.s.	n.s.	n.s.	n.s.
15	D	No C_q_	n.s.	n.s.	n.s.	n.s.
16	D	35.23	n.s.	35.34	No C_q_	n.s.
17	E	No C_q_	n.s.	n.s.	n.s.	n.s.
18	E	No C_q_	n.s.	n.s.	n.s.	n.s.
Samples tested (n)		18	1	3	3	2

n.s.: no sample.

## Supplementary Materials

The following are available online at https://www.mdpi.com/2306-7381/7/4/201/s1, Figure S1: ORF27/gp48 nucleotide and amino acid sequence alignment; Table S1: Pairwise-distance matrix of ORF27 nucleotide sequences; and Table S2: Pairwise-distance matrix of gp48 amino acid sequences.
