# Supplementary figures and images for: Field Observations and Genetic Characterization of Sheep-Associated Malignant Catarrhal Fever in Egypt, 2018
Source: Vet Sci. 2020 Dec 11;7(4):201. doi: 10.3390/vetsci7040201 (PMC7768452; doi:10.3390/vetsci7040201)

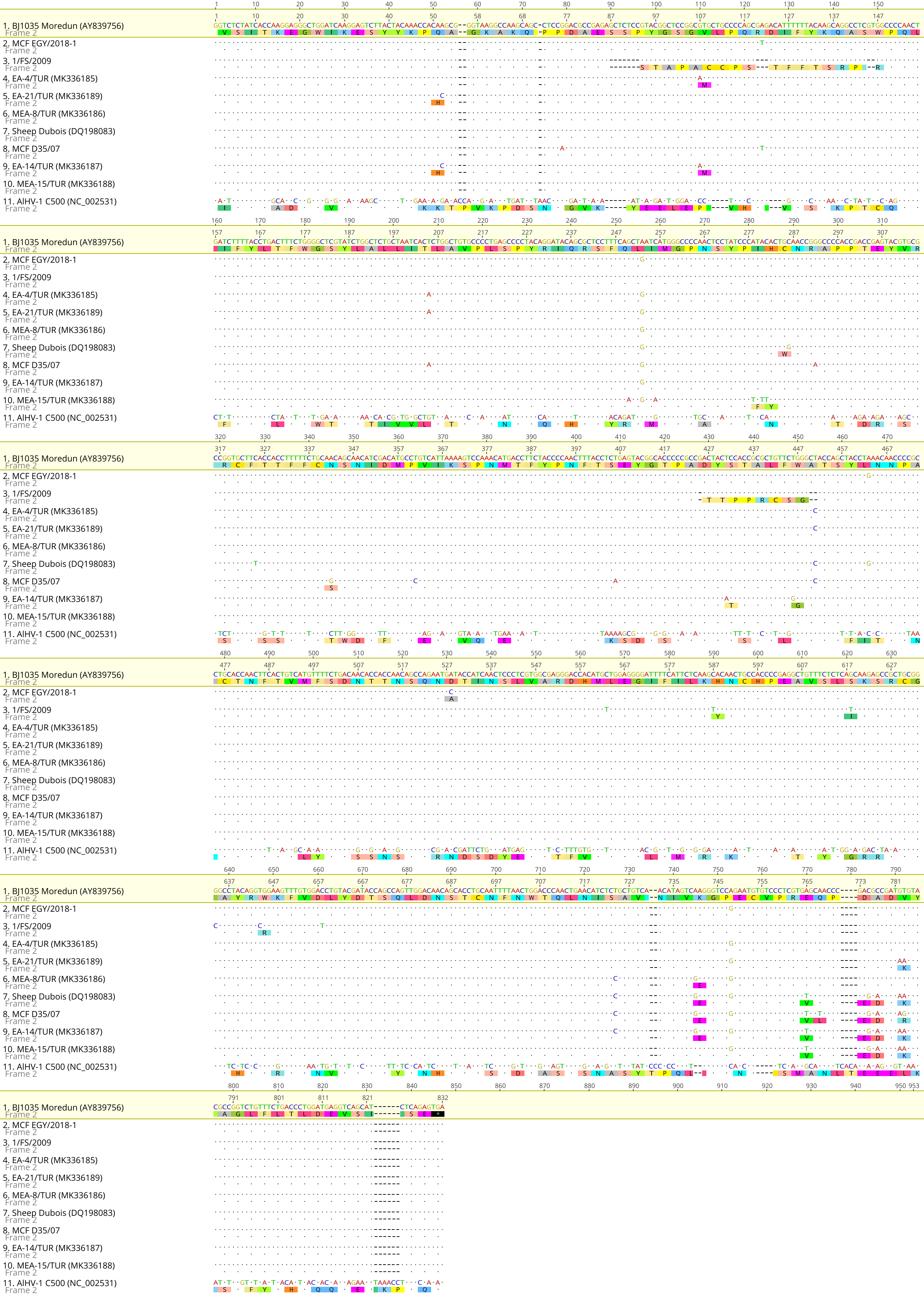

Supplement: Supplementary file 1 [file vetsci-07-00201-s001.zip › Figure S1.pdf]
